# Challenges in Providing Gynecological Procedures in Primary Care: A Survey of Canadian Academic Family Physicians

**DOI:** 10.1089/whr.2024.0098

**Published:** 2025-01-24

**Authors:** Parisa Rezaiefar, Douglas Archibald, Monisha Kabir, Susan Humphrey-Murto

**Affiliations:** ^1^ Reproductive and Sexual Health, Origyns Medical Clinic, Ottawa, Ontario, Canada.; ^2^ Department of Family Medicine, Faculty of Medicine, University of Ottawa, Ottawa, Ontario, Canada.; ^3^ Bruyère Research Institute, Ottawa, Ontario, Canada.; ^4^ Faculty of Medicine, University of Ottawa, Ottawa, Ontario, Canada.; ^5^ Research Support Unit, Department of Innovation in Medical Education, University of Ottawa, Ottawa, ON, Canada.

**Keywords:** physicians, family, access to primary care, gynecologic procedures, women’s health

## Abstract

**Purpose:**

Globally, there is a lack of access to health care providers who offer gynecological procedures. Understanding the practice patterns of academic family physicians (AFPs) and whether additional training impacts the provision of care is critical. This study surveys the practice patterns of AFPs regarding gynecological procedures offered, identifies barriers, and explores the impact of additional training.

**Methods:**

We circulated an anonymous, cross-sectional survey to all 17 family medicine programs across Canada, receiving responses from 71 AFPs. We computed descriptive statistics and bivariate associations.

**Results:**

A total of 71 respondents from five universities participated. Most participants (97.2%) performed Papanicolaou (Pap) smears; 67.6% provided intrauterine device (IUD) insertion, and only 54.9% offered endometrial biopsy. Numbers decreased significantly for routine pessary care (29.5%), punch biopsy of the vulva (15.5%), and pessary fitting (5.6%). Eighteen participants (26.9%) had received enhanced skills training with a certificate of added competence (CAC), of which 55.6% were in women’s health. CAC holders in women’s health provided IUD insertions (100% vs. 67.3%; *p* = 0.049, V = 0.28) and endometrial biopsies (90.0% vs. 53.1%; *p* = 0.036, V = 0.28) at higher rates than general AFPs. Frequently cited barriers to offering gynecological procedures included lack of knowledge, procedural skills, and insufficient patient volumes to maintain competence. During the COVID-19 pandemic, 44% of respondents reported reducing or ceasing to provide Pap smears.

**Conclusions:**

Many AFPs in Canada do not provide essential gynecological procedures. This impacts patient access and the training of the next generation of family physicians and thus requires innovative strategies to address the persistent procedural skills educational gap for trainees.

## Introduction

Lack of access to office-based gynecological procedures (OBGPs) increases health inequity and has adverse health^1^ and economic consequences.^2^ Despite recognition of these issues, women and individuals assigned female at birth continue to experience barriers to accessing such services globally.^3–9^ Furthermore, the COVID-19 pandemic has increased wait times for nearly all health care services internationally.^10–12^

Family physicians constitute approximately half of the physician workforce in the United States and Canada and play a crucial role in providing OBGPs.^13,14^ The extent to which the COVID-19 pandemic has widened pre-existing gaps in access to OBGPs is unclear, except for Papanicolaou (Pap) smears for cervical cancer screening. One Canadian study reported decreased Pap smear screening during the pandemic, particularly for individuals living on a First Nations reserve.^15^

The family physician’s profile as a proceduralist differs globally;^16^ however, Canada and the United States are two countries with the most similarities in the OBGP competencies expected of graduating family physicians. In Canada, mandatory OBGP skills are referred to as list A (“all graduates will be prepared to perform the procedures in list A”).^17,18^ This list includes Pap smears, intrauterine device (IUD) insertion for long-term reversible contraception, and endometrial aspiration biopsy for endometrial cancer detection.^17,18^ In the United States, category A or core procedures are those in which “all family medicine residency programs must provide training,” and more specifically, category A1 refers to procedures which “all residents must be able to perform independently by graduation.”^16,19^ These procedures include Pap smear, vulvar biopsy, Bartholin’s cyst management, removal of a cervical polyp, endometrial biopsy, IUD insertion, and fine needle aspiration of breast.^16,19^ The Canadian list was updated in 2021 to include vaginal pessary fitting and routine care for the management of pelvic organ prolapse and stress urinary incontinence in response to the increased prevalence of these conditions at approximately 50% of parous women in the aging population.^17^ The latest national data from 2004 reported that few Canadian family physicians provided OBGPs.^13^ While 77% provided Pap smears, 35% inserted IUDs, and only 15% performed endometrial biopsies.^13^ Similar data are reported in the United States, where only 25% of family medicine graduates reported performing endometrial biopsies and 41% inserted IUDs.^20^ As for pessary fitting and routine care, most studies have focused on the provision of these procedures by gynecologists and urogynecologists.^21–23^ The three multidisciplinary surveys in the medical, nursing, midwifery, and physiotherapy contexts suggest that few family physicians and general practitioners provide pessary fitting and routine care due to a lack of training.^24–26^

The shortage of skilled primary care providers is a well-reported barrier to patients’ access to OBGPs.^27,28^ Lack of clinical exposure during residency training has been highlighted, with 19% of 1803 Canadian family medicine residents reporting minimum or no exposure to office-based procedural skills upon residency completion.^29^ Other obstacles to acquiring adequate skills include limited opportunities to practice and short residency programs.^30,31^ The challenges in acquiring adequate skills become further compounded upon entering clinical practice due to a lack of time, support staff, and financial incentives, leading to an inability to maintain skills and provide OBGP procedures.^32,33^

Family medicine residency graduates report an increased likelihood of providing OBGPs upon receiving additional training.^27,34,35^ However, strategies outside of increasing the length of training may be required. For example, only one-half of American graduates from 3-year family medicine residency programs provide one or more gynecological procedures despite stated “adequate training.”^20^ Within the Canadian context, it is unclear if the certificate of added competence (CAC) for enhanced skills in women’s health or low-risk obstetrics, which involves an additional year of training, has improved access to OBGPs.^36^ Despite the upward trends toward obtaining a CAC, 70% of family physicians do not pursue additional clinical training.^13,37^

Academic family physicians (AFPs) deliver most of the postgraduate curricula to medical students, residents, and fellows during the core or elective training by providing formal teaching and supervision in ambulatory care settings.^38^ An American survey of full-time faculty from general internal medicine and family practice in nine states found that only a minority were prepared to precept any gynecological procedures except for the Pap smear.^39^ Because AFPs’ knowledge, skills, and practice patterns may impact trainees’ competence and future practice, a better understanding of AFPs practice patterns may provide insights into the persistent lack of improvement in providing gynecological procedures in primary care despite an increase in competency-based training.

Most published surveys are outdated or focus on practicing primary care providers rather than those providing training to learners and future family physicians. Therefore, the objectives of this study were to explore the practice patterns of Canadian AFPs for gynecological procedures deemed mandatory for graduating family physicians, identify barriers, and explore the impact of additional training. Such knowledge is essential to developing effective strategies to enhance family medicine training by optimizing the clinical environment in which trainees learn, which may in turn improve patient access. We also explored the impact of the COVID-19 pandemic on providing OBGPs, as such events may occur in the future.

## Methods

We developed a cross-sectional survey using elements of two previously published surveys,^13,32^ and validated it using the seven-step process delineated by Artino et al.^40^ Content validity was ensured by an initial Medline, Education Source (*via* EBSCO), and Google Scholar search to identify core OBGPs competencies within the scope and expected of family physicians.^16,17,19,41–43^ We also searched for documented barriers^27,28,32^ that providers encounter when delivering gynecological procedures. We then interviewed six expert university-affiliated family physicians who perform, supervise, and teach OBGPs across three Canadian institutions. We sought their opinions on OBGPs for inclusion in the survey. The resulting list included Pap smear, IUD insertion, endometrial biopsy, vaginal pessary fitting, and routine care corresponding to five gynecological procedures from the College of Family Physicians of Canada’s priority list that are integral to providing comprehensive care.^19,42,44–49^ Interviewees recommended that the punch biopsy of the vulva be added to the list because it is a core gynecological procedural skill in the United States but not in Canada.^16,19^ The data on the access to the punch biopsy of the vulva in primary care are lacking and vulvar disease is underrecognized in primary care.^50^

The interviewees suggested an exhaustive list of perceived barriers from five domains: knowledge, skills, attitude, patient preferences, and environmental factors. Three focus groups were subsequently used to reduce the number of items and refine the survey. Focus groups included seven AFPs from one university representing the target survey respondents. During each focus group, attendees completed the survey. They provided verbal feedback on the ease of use, visual design, language clarity, comprehension, completeness, and relevance of selected procedures and perceived barriers. Finally, the survey was pilot-tested *via* SurveyMonkey® with an additional eight AFPs representing the target survey respondents (*i.e.*, AFPs who hold an academic appointment and spend at least 20% of their time practicing family medicine). We incorporated feedback from the focus groups and pilot-testing into the final survey.

The Bruyère Continuing Care (#M16-20-033), Ottawa Health Sciences Network (#20200069-01H), and University of Ottawa (#H-10-21-7366) research ethics boards approved this study. Respondents indicated their consent to participate in the research electronically within the survey.

### Data collection

The final anonymous survey had 14 questions (https://www.surveymonkey.ca/r/F6Z3KWW), with the first question designed to exclude AFPs with less than 20% time in clinical practice. We explored practice patterns by assessing participants’ comfort in performing OBGPs, the frequency of OBGP provision in a typical year pre-pandemic and during the pandemic, and the individuals to whom procedures were delegated (*e.g.*, gynecologists, nurses, and nurse practitioners). One question offered 18 barriers to performing OBGPs from four domains (knowledge, skill, attitude, and systemic), all of which could be selected, and a free text option for additional barriers. Participant demographics included gender, years in practice, program affiliation, completion of additional training, practice type, and population served.

The postgraduate office of the primary investigator’s institution sent the survey invitation to postgraduate directors at all family medicine departments in Canada (*n* = 17) for dissemination within their institutions. Reminder emails were sent 1 and 3 weeks after the initial email to improve the response rate.^51^ Data collection occurred between January and June 2022. Respondents voluntarily entered a draw for three $100 gift certificates *via* email following survey completion.

### Data analysis

Descriptive statistics and bivariate associations using Fisher’s Exact test (two-sided *p*-values and effect sizes using Cohen’s V reported) were computed for the appropriate study variables using IBM SPSS Statistics (version 28.0.1.1). There were no modifications to variables, no weighting of items, and no propensity scores used.

## Results

Ninety-one participants across five of 17 (29%) family medicine departments (located across Canada in Alberta, Quebec, Ontario, and Nova Scotia) completed at least one survey question, representing 1.7% across the five family medicine departments (*n* = 5,445 total faculty members). Twenty (22.0%) respondents were excluded based on their response to the first question, leaving 71 respondents. Of these, four respondents did not complete the demographics questions, and one also did not complete the questions on barriers. The responses from these participants (*n* = 4) were included for all questions they completed. The survey response rate could not be calculated as postgraduate directors declined to share their faculty members’ email addresses and the total number of AFPs.

Self-reported female physicians represented 68.7% of the respondents; 26.9% identified as male and 4.5% of respondents preferred not to disclose their gender. Thirty-one percent of respondents reported more than 21 years in practice, while 17.9% were recent graduates, defined as 5 years or less in practice. Half of the participants were mid-career AFPs (6–10 years: 25.4%; 11–20 years: 25.4%). More than half of the respondents were affiliated with the University of Ottawa at 55.2%, followed by Dalhousie University at 34.3%, the University of Alberta at 6.0%, and McGill University and the University of Toronto at 1.5% each.

The completion of a third year in CAC was reported by 26.9% of participants. Notably, enhanced skills in women’s health or low-risk obstetrics constituted 55.6% of CACs. Most participants reported practicing in a group at 84.4%, with 12.5% in solo practice. More than half of respondents (52.5%) served urban/suburban populations, followed by the inner city (16.2%), small town (20.0%), and rural (18.6%). A minority served geographically isolated/remote communities (4.3%) and mixed rural/small cities (1.4%). Respondent demographics are presented in Table 1.

**Table 1.** Respondent Demographics

Characteristic (row %)	*N* (%)
Gender (*N* = 67)	
Female	46 (68.7)
Male	18 (26.9)
Prefer not to disclose	3 (4.5)
Years in practice (*N* = 67)	
0–5 years	12 (17.9)
6–10 years	17 (25.4)
11–20 years	17 (25.4)
>21 years	21 (31.3)
Family Medicine Program affiliation (*N* = 67)	
University of Ottawa	37 (55.2)
Dalhousie University	23 (34.3)
University of Alberta	4 (6.0)
McGill University	1 (1.5)
University of Toronto	1 (1.5)
Completion of third-year (PGY3) enhanced skills program (*N* = 67)	
No	49 (73.1)
Yes	18 (26.9)
Third-year enhanced skills program (*N* = 18)^a^	
Women’s health or low-risk obstetrics	10 (55.6)
Other	8 (44.4)
Population served by family medicine practice^b^ (*N* = 99)	
Inner city	16 (16.2)
Urban/suburban	52 (52.5)
Small town	14 (20.0)
Rural	13 (18.6)
Geographically isolated/remote	3 (4.3)
Mixed rural/small city	1 (1.4)
Organization of family medicine practice (*N* = 64)	
Solo practice	8 (12.5)
Group practice	54 (84.4)
Other	2 (3.1)

^a^ The number of respondents for this question does not align with the total number of responses as this question only applied to those who answered “Yes” to the prior question.

^b^ The number of respondents does not align with the total number of responses as respondents were able to select all applicable options (*N* = 99 total responses).

While 97.2% of participants reported performing Pap smears, provision dropped to 67.6% for IUD insertion, 54.9% for endometrial biopsy, 29.5% for routine pessary care, 15.5% for punch biopsy of the vulva, and 5.6% for pessary fitting (Table 2). We observed a similar pattern for respondents’ comfort level (Table 3).

**Table 2.** Frequency of Performing Office-Based Gynecological Procedures in a Typical Year Prior to the COVID-19 Pandemic (*N* = 71)

	*N* (%)	
Procedure	Does not perform procedure (0/month)	Performs procedure (≥1 month)
Pap smear	2 (2.8)	69 (97.2)
Intrauterine device insertion	23 (32.4)	48 (67.6)
Endometrial aspiration/biopsy	32 (45.1)	39 (54.9)
Punch biopsy of vulva	60 (84.5)	11 (15.5)
Pessary fitting	67 (94.4)	4 (5.6)
Routine pessary care^a^	50 (70.4)	21 (29.6)

^a^ *i.e.*, removing/re-inserting the pessary and examining the vagina.

Pap, Papanicolaou.

**Table 3.** Comfort Level with Performing Office-Based Gynecological Procedures (*N* = 71)

	*N* (%)			
Procedure	Very uncomfortable	Uncomfortable	Comfortable	Very comfortable
Pap smear	2 (2.8)	1 (1.4)	0 (0)	68 (95.8)
Intrauterine device insertion	11 (15.5)	10 (14.1)	13 (18.3)	37 (52.1)
Endometrial aspiration/biopsy	12 (16.9)	18 (25.4)	15 (21.1)	26 (36.6)
Punch biopsy of vulva	18 (25.4)	30 (42.3)	15 (21.1)	8 (11.3)
Pessary fitting	33 (46.5)	31 (43.7)	3 (4.2)	4 (5.6)
Routine Pessary Care^a^	17 (23.9)	18 (25.4)	22 (31.0)	14 (19.7)

^a^ *i.e.*, removing/re-inserting the pessary and examining the vagina.

Pap, Papanicolaou.

Forty-four percent of participants reported a moderate to significant reduction or complete cessation of performing Pap smears during the COVID-19 pandemic (Table 4). Approximately 20% of respondents reported a reduction or complete cessation of offering all other OBGPs.

**Table 4.** Change in Performing Office-Based Gynecological Procedures Since the COVID-19 Pandemic (*N* = 71)

	Change in performing procedure, *N* (%)				
Office-based gynecological procedure	None to minimal change (<30%)	Moderate reduction (30%–50%)	Significant reduction (<50%)	Stopped doing all together (100%)	Total reduction or cessation
Pap smear	40 (56.3)	25 (35.2)	6 (8.5)	0 (0)	31 (43.7)
Intrauterine device (IUD) insertion	57 (80.3)	8 (11.3)	4 (5.6)	2 (2.8)	14 (19.7)
Endometrial aspiration/biopsy	54 (76.1)	6 (8.5)	6 (8.5)	5 (7.0)	17 (23.9)
Punch biopsy of vulva	56 (78.9)	1 (14)	6 (8.5)	8 (11.3)	15 (21.2)
Pessary fitting	57 (80.3)	0 (0)	4 (5.6)	10 (14.1)	14 (19.7)
Routine pessary care^a^	55 (77.5)	5 (7.0)	3 (4.2)	8 (11.3)	16 (22.5)

^a^ *i.e.,* removing/re-inserting the pessary and examining the vagina.

Supplementary Table S1 reports bivariate associations between respondent characteristics and OBGPs performed. There was no statistical difference between general AFPs and all CAC holders (Pap smear: *p* = 0.47, V = 0.09; IUD insertion: *p* = 0.78, V = 0.05; endometrial biopsy: *p* = 0.59, V = 0.07; punch biopsy of the vulva: *p* = 0.12, V = 0.22; routine pessary care: *p* = 1.0, V = 0.03; and pessary fitting: *p* = 0.29, V = 0.13). However, compared with general AFPs, more participants with a CAC in women’s health or low-risk obstetrics reported providing IUD insertion (67.3% vs. 100%; *p* = 0.049, V = 0.28), endometrial biopsy (53.1% vs. 90.0%; *p* = 0.038, V = 0.28), and punch biopsy of the vulva (10.2% vs. 40.0%; *p* = 0.036, V = 0.31). These comparisons were statistically significant with moderate effect sizes. Routine pessary care provision was similarly low for general AFPs and CAC holders in women’s health or low-risk obstetrics (30.6% vs. 30.0%; *p* = 1.0; V = 0.01). Only 4.1% of the general AFPs and 10.0% of the 10 CAC holders in women’s health or low-risk obstetrics reported providing pessary fitting. There were no meaningful statistically significant differences for other comparisons.

**FIG. 1.**
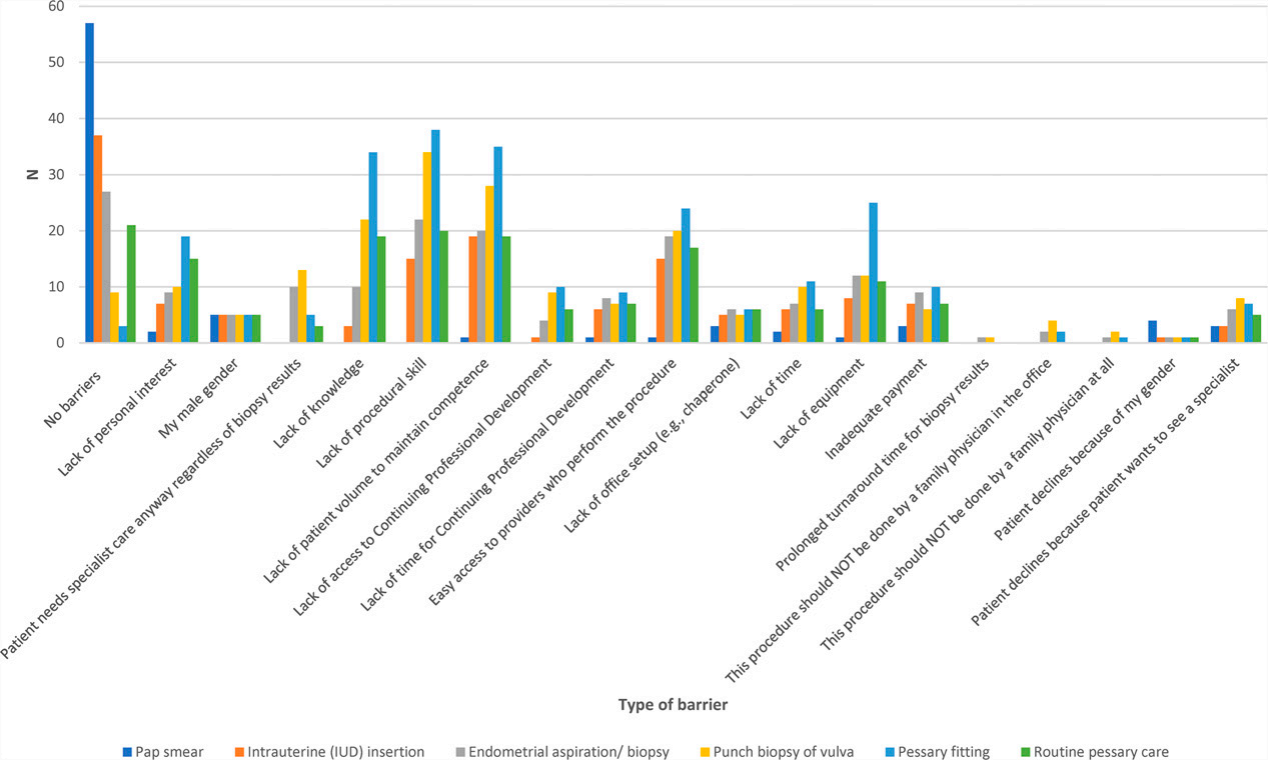
Barriers impacting family physicians’ ability to perform gynecologic procedures (*N* = 67).^a^ ^a^Number of respondents (*N* = 67) does not align with the total number of barriers reported as respondents were able to select all applicable options.

The most frequently cited barriers to performing all procedures except Pap smears were a lack of knowledge, procedural skills, sufficient patient volumes to maintain competence, and easy access to providers who perform the procedure (Fig. 1). Male gender, office set-up (*e.g.*, access to a chaperone), and inadequate payment were reported as barriers by less than 14.9% of participants. Fewer than 11.9% reported that patients preferred to see a specialist, and none reported prolonged turnaround time for the biopsy results (≤1.5%). Similarly, across all procedures except for Pap smear (6.0%), almost no respondents reported patient refusal to have the procedure done because of the respondents’ gender (1.5%). As for physicians’ attitudes, lack of personal interest was varied, with the lowest reported for Pap smear (3.0%) followed by IUD insertion (10.4%), endometrial biopsy (13.4%), punch biopsy of the vulva (14.9%), routine pessary care (22.4%), and pessary fitting (28.4%). However, few respondents (≤6.0%) cited that these procedures should not be performed in a family physician’s office or by a family physician.

IUD insertion was most frequently referred to another family physician (41.4%), followed by gynecologists at 27.1% (Table 5). Conversely, respondents referred to gynecologists more frequently than family physician colleagues for endometrial biopsy (42.9% vs. 25.7%), pessary fitting (61.4% vs. 40.0%), routine pessary care (47.1% vs. 31.5%), and punch biopsy of the vulva (70.0% vs. 31.4%). Respondents rarely (2.9%–5.7%) referred patients to other primary care providers (*e.g.*, nurses, nurse practitioners, or physiotherapists), except for pessary fitting (10.0%) and routine pessary care (18.6%).

**Table 5.** Delegation of Office-Based Gynecological Procedure Completion (*N* = 70)^a^

	Office-based gynecological procedure, *N* (%)					
Delegate	Pap smear	Intrauterine device (IUD) insertion	Endometrial aspiration/biopsy	Punch biopsy of vulva	Pessary fitting	Routine pessary care^b^
Family physician colleague in my office	6 (8.6)	17 (24.3)	12 (17.1)	10 (14.3)	8 (11.4)	6 (8.6)
Nurse/nurse practitioner in my office	10 (14.3)	1 (1.4)	0 (0)	0 (0)	1 (1.4)	4 (5.7)
Specialty clinic run by family physicians	4 (5.7)	12 (17.1)	6 (8.6)	12 (17.1)	20 (28.6)	16 (22.9)
An OB/GYN	6 (8.6)	19 (27.1)	30 (42.9)	49 (70.0)	43 (61.4)	33 (47.1)
Other (*e.g.*, women’s health clinic, nurse-run specialty clinic, procedure clinic, urogynecologist, and physiotherapist)	2 (2.9)	4 (5.7)	4 (5.7)	4 (5.7)	7 (10.0)	13 (18.6)
Do not delegate (*i.e.*, respondent performs or supervises learner performing procedure)	51 (72.9)	35 (50.0)	25 (35.7)	13 (18.6)	7 (10.0)	10 (14.3)

^a^ *i.e.*, removing/re-inserting the pessary and examining the vagina.

^b^ The number of respondents (*N* = 70) does not align with the total number of delegates reported as respondents were able to select all applicable options.

OB, obstetrics; GYN, gynecology.

## Discussion

The findings of this cross-sectional survey are significant, revealing that a considerable number of AFPs in Canada do not provide essential gynecological procedures. We report the alarming lack of primary care physicians who provide vaginal pessary fitting, routine pessary care, and punch biopsy of the vulva. We also provide a comprehensive list of barriers to providing OBGPs in the academic environment where future family physicians are trained.

It is encouraging that a higher percentage of AFPs report performing Pap smear (97.2%), IUD insertion (67.6%), and endometrial biopsy (54.9%) compared with previously reported literature among Canadian family physicians and much higher than reported by American AFPs.^13,52^ However, it is concerning that very few AFPs provide pessary fitting, routine care, and punch biopsy of the vulva. Given the alarming increase in wait times to see gynecologists, with the average wait time of about 15.7 weeks in 2022 compared with 8.7 weeks in 2014 in Canada,^53^ the lack of timely access to gynecological care may exacerbate already reported health disparities among women and those assigned female at birth.

Our study suggests that enhanced training in women’s health and low-risk obstetrics improves the provision of some procedures but not others. All AFPs with CACs in women’s health or low-risk obstetrics reported providing IUD insertion, and 90% reported providing endometrial biopsy. The lack of training on inserting the pessary during medical postgraduate training has been cited as a reason for family physicians not providing pessary fitting and routine care.^24^

Until our study, the limited data on the provision of vulvar biopsy have focused on dermatologists and gynecologists,^54,55^ and have reported the need for more training in vulva biopsy to improve their willingness to perform this procedure.^54,55^ The punch biopsy of skin is a core procedural skill for graduating family physicians in Canada and the United States.^17,18^ It can be performed on the vulva to differentiate vulvar cancer and precancerous changes from inflammatory or more common vulva conditions that affect one in five women in their lifetime, leading to more expeditated care.^56^ Yet, a punch biopsy of the vulva is not a core gynecological procedure for graduating family medicine trainees. This might explain our finding that despite the additional training, only 40% of CAC respondents provided punch biopsy of the vulva. The provision of pessary fitting, and routine pessary care was similar to that of the general AFPs. Therefore, a comprehensive evaluation of the enhanced skills program in women’s health and low-risk obstetrics and its graduates is required to understand how additional training translates to competence and willingness to provide OBGP in primary care.

Over 30% of respondents reported a lack of sufficient patient volume to maintain competence, knowledge, and procedural skills as barriers, particularly for pessary fitting and punch biopsy of the vulva. Based on the prevalence of pelvic organ prolapses,^49^ stress urinary incontinence,^57^ and vulva complaints^49^ in the general population, it is unlikely that AFPs do not see patients who require these procedures. Several factors may explain the perceived lack of prevalence reported by participants. For example, on average, family physicians manage 3.3 problems per adult patient per visit.^58^ Genitourinary and vulva concerns are associated with shame and poor psychological health.^59,60^ Therefore, patients and providers may not prioritize these conditions during short visits, leading to further under-recognition and loss of physician skills.

Our findings highlight the negative impacts of the COVID-19 pandemic on access to gynecological procedures. The significant reduction in cervical cancer screening noted in our study is consistent with other Canadian data and is concerning.^15^ Superimposed on the existing pre-pandemic inequity and the decline in cervical cancer screening among eligible patients between 2005 and 2019 reported by the National Cancer Institute, decreases in cervical cancer screening may lead to an increased resurgence of cervical cancer,^61,62^ the fourth leading cause of cancer mortality globally.^63^ Similarly, the reduction in the provision of endometrial biopsies during the COVID-19 pandemic is concerning given that delay in diagnosis of endometrial cancer has significant negative impacts on patient survival.^64^

The increasing demand for gynecological procedures cannot be met by gynecologists alone.^37^ The lack of access to OBGPs in academic primary care demonstrated in our study requires an urgent, robust, and multipronged approach to address the gap in patient access and trainees’ education. For example, it is unclear why our study participants underutilized other allied health professionals when referring patients. In other countries, midwives, nurses, and physiotherapists provide pessary fitting and routine care.^65^ IUD insertion is also performed by nurses^66^ and midwives globally.^67,68^ Strategies should include closer attention to and examination of all available skilled providers in primary care including but not limited to CAC holders, such as nurses, nurse practitioners, and physiotherapists.^39,69^ Implementing a centralized referral system that includes all providers with skills in gynecological procedures could help delegate care to appropriate providers, reduce wait time for gynecologists, and improve patient access.^70,71^

Finally, the lack of skilled AFPs providing gynecological procedures reported in our study hinders the knowledge and skills acquisition of future family physicians, something that has not been adequately explored previously. This educational gap requires strategies that train learners and practicing physicians simultaneously. Investing in capacity within AFPs and other allied health professionals is crucial for trainees to gain the knowledge, skills, and attitude necessary to provide OBGPs upon graduation. Educators can address the need for more capacity among AFPs by offering targeted continuing professional development to community family physicians and other allied health professionals,^72,73^ faculty development, and mentoring to AFPs who require additional experience to gain confidence.^74^ Innovative educational strategies such as virtual teaching and gamification engage AFPs and trainees in learning, an advantage for ever-growing family medicine trainees.^75^ Such innovations allow for skills development^76^ through repetition, address the lack of patient volume, and improve confidence.^77^ Our team will be launching virtual teaching and gamification tools for access by all local primary care providers.

Limitations for this study include our inability to calculate a response rate across all AFPs in Canada, a low response rate across the five universities from which AFPs completed our survey, and the overrepresentation of our own institution in our results. Our very low response rate can be explained by the timing of our study. In 2022, family physician burnout due to the COVID-19 pandemic was a serious concern.^78^ Family physicians had limited time to spare as they had started to tackle missed routine preventative care such as immunizations.^79^ Therefore, due to concerns about physicians’ wellness, the College of Family Physicians of Canada and several departments of family medicine declined to distribute the survey. However, evidence suggests that response rates may not be as strongly correlated with survey quality or representativeness as once thought.^80,81^ Moreover, this survey included representation from central, east, and west Canadian provinces, and a French-speaking province, thus covering key geographical areas of Canada. Finally, our institution is comparable in size with others across the country. Therefore, the results of our study may be transferrable to other similar institutions. Future research could explore the effects of training more early to mid-career AFPs who could then train the next generation. This study is limited in its power by the small number of participants, especially in the subgroups of CAC holders with enhanced skills in women’s health or low-risk obstetrics. Lastly, the use of a survey for data collection may have contributed to self-report bias. However, the percentage of respondents who reported performing an OBGP followed a similar trend as those who felt comfortable performing each procedure. This finding is consistent with evidence suggesting that physicians’ confidence impacts their likelihood of providing such services,^82,83^ which lends some credibility to the data. Future studies can consider billing codes for more objective measures of practice patterns.

## Conclusion

The lack of AFPs in Canada who provide essential gynecological procedures and the interruption of these services during the COVID-19 pandemic is alarming for equitable health care access and curriculum delivery to future family physicians. This aligns with the global issues identified. We urge policymakers and educators to consider innovative strategies to improve patient access by implementing a centralized referral system that includes all primary care providers and enhancing the education of gynecological procedures in primary care, where care is more cost-effective, closer to patients’ geographic location, and timelier.

## Data Availability

The dataset used in this study is available upon request from the corresponding author.

## Abbreviations Used

AFPs: Academic family physicians
CAC: Certificate of added competence
IUD: Intrauterine device
OBGPs: office-based gynecological procedures
Pap smears: Papanicolaou smears
