# Supplementary material for: Challenges in Providing Gynecological Procedures in Primary Care: A Survey of Canadian Academic Family Physicians
Source: Womens Health Rep (New Rochelle). 2025 Jan 24;6(1):102–12. doi: 10.1089/whr.2024.0098 (PMC11839541; doi:10.1089/whr.2024.0098)
Supplement: Supplementary Data [file whr.2024.0098_supp_tables1.pdf]

Supplemental Table 1. Bivariate associations using Fisher's Exact test (two-sided p-values reported) between office-based gynecological procedures performed and respondent characteristics.

	Pap smear				Intrauterine device (IUD) insertion				Endometrial aspiration/biopsy				Punch biopsy of vulva				Pessary fitting				Routine pessary care			
**Characteristic (row %)**	Not performed (0/month)	Performed (≥1/month)	*p*	Effect size (Cramer's V)[1]	Not performed (0/month)	Performed (≥1/month)	*p*	Effect size (Cramer's V)	Not performed (0/month)	Performed (≥1/month)	*p*	Effect size (Cramer's V)	Not performed (0/month)	Performed (≥1/month)	*p*	Effect size (Cramer's V)	Not performed (0/month)	Performed (≥1/month)	*p*	Effect size (Cramer's V)	Not performed (0/month)	Performed (≥1/month)	*p*	Effect size (Cramer's V)
Gender			0.53	0.10 (weak)			0.72	0.15 (weak)			0.37	0.20 (moderate)			0.28	0.18 (weak)			0.15	0.28 (moderate)			1.0	0.03 (negligible)
Male	1 (5.6)	17 (94.4)			6 (33.3)	12 (66.7)			8 (44.4)	10 (55.6)			17 (94.4)	1 (5.6)			18 (100)	0 (0)			12 (66.7)	6 (33.3)		
Female	1 (2.2)	45 (97.8)			15 (32.6)	31 (67.4)			22 (47.8)	24 (52.2)			38 (82.6)	8 (17.4)			43 (93.5)	3 (6.5)			32 (69.6)	14 (30.4)		
Prefer not to disclose	0 (0)	3 (100)			0 (0)	3 (100)			0 (0)	3 (100)			2 (66.7)	1 (33.3)			2 (66.7)	1 (33.3)			2 (66.7)	1 (33.3)		
Years in practice			0.68	0.18 (weak)			0.29	0.24 (moderate)			0.62	0.17 (weak)			0.55	0.18 (weak)			0.03	0.37 (moderate)			0.91	0.10 (weak)
0-5 years	0 (0)	12 (100)			2 (16.7)	10 (83.3)			4 (33.3)	8 (66.7)			9 (75.0)	3 (25.0)			12 (100)	0 (0)			9 (75.0)	3 (25.0)		
6-10 years	1 (5.9)	16 (94.1)			8 (47.1)	9 (52.9)			9 (52.9)	8 (47.1)			14 (82.4)	3 (17.6)			17 (100)	0 (0)			12 (70.6)	5 (29.4)		
11-20 years	1 (5.9)	16 (94.1)			6 (35.3)	11 (64.7)			9 (52.9)	8 (47.1)			16 (94.1)	1 (5.9)			17 (100)	0 (0)			12 (70.6)	5 (29.4)		
>21 years	0 (0)	21 (100.0)			5 (23.8)	16 (76.2)			8 (38.1)	13 (61.9)			18 (85.7)	3 (14.3)			17 (81.0)	4 (19.0)			13 (61.9)	8 (38.1)		
Family Medicine Program affiliation			0.31	0.24 (moderate)			0.27	0.31 (moderate)			0.002[a]	0.47 (relatively strong)			0.29	0.34 (moderate)			0.21	0.49 (relatively strong)			0.46	0.27 (moderate)
University of Alberta	0 (0)	4 (100)			0 (0)	4 (100)			0 (0)	4 (100)			4 (100)	0 (0)			4 (100)	0 (0)			3 (75.0)	1 (25.0)		
University of Saskatchewan	0 (0)	1 (100)			0 (0)	1 (100)			0 (0)	1 (100)			0 (0)	1 (100)			0 (0)	1 (100)			0 (0)	1 (100.0)		
University of Toronto	0 (0)	1 (100)			0 (0)	1 (100)			0 (0)	1 (100)			1 (100)	0 (0)			1 (100)	0 (0)			1 (100)	0 (0)		
University of Ottawa	0 (0)	37 (100)			10 (27.0)	27 (73.0)			13 (35.1)	24 (64.9)			30 (81.1)	7 (18.9)			35 (94.6)	2 (5.4)			23 (62.2)	14 (37.8)		
McGill University	0 (0)	1 (100)			0 (0)	1 (100)			0 (0)	1 (100)			1 (100)	0 (0)			1 (100)	0 (0)			1 (100)	0 (0)		
Dalhousie University	2 (8.7)	21 (91.3)			11 (47.8)	12 (52.2)			17 (73.9)	6 (26.1)			21 (91.3)	2 (8.7)			22 (95.7)	1 (4.3)			18 (78.3)	5 (21.7)		
Completion of third-year (PGY3) enhanced skills program (all)			0.47	0.09 (negligible)			0.78	0.05 (negligible)			0.59	0.07 (negligible)			0.12	0.22 (moderate)			0.29	0.13 (weak)			1.0	0.03 (negligible)
No	1 (2.0)	48 (98.0)			16 (32.7)	33 (67.3)			23 (46.9)	26 (53.1)			44 (89.8)	5 (10.2)			47 (95.9)	2 (4.1)			34 (69.4)	15 (30.6)		
Yes	1 (5.6)	17 (94.4)			5 (27.8)	13 (72.2)			7 (38.9)	11 (61.1)			13 (72.2)	5 (27.8)			16 (88.9)	2 (11.1)			12 (66.7	6 (33.3)		
No PGY3 vs. completion of PGY3 in women's health or low-risk obstetrics			1.0	0.06 (negligible)			0.049[a]	0.28 (moderate)			0.038[a]	0.28 (moderate)			0.036[a]	0.31 (moderate)			0.43	0.10 (weak)			1.0	0.01 (negligible)
No PGY3 training	1 (2.0)	48 (98.0)			16 (32.7)	33 (67.3)			23 (46.9)	26 (53.1)			44 (89.8)	5 (10.2)			47 (95.9)	2 (4.1)			34 (69.4)	15 (30.6)		
PGY3 in women's health or low-risk obstetrics	0 (0)	10 (100)			0 (0)	10 (100)			1 (10.0)	9 (90.0)			6 (60.0)	4 (40.0)			9 (90.0)	1 (10.0)			7 (70.0)	3 (30.0)		
Third-year enhanced skills program			0.44	0.27 (moderate)			0.007[a]	0.69 (strong)			0.01[a]	0.66 (strong)			0.31	0.31 (moderate)			1.0	0.04 (negligible)			0.63	0.20 (moderate)
Women's health or low-risk obstetrics	0 (0)	10 (100)			0 (0)	10 (100)			1 (10.0)	9 (90.0)			6 (60.0)	4 (40.0)			9 (90.0)	1 (10.0)			7 (70.0)	3 (30.0)		
Other	1 (5.6)	7 (87.5)			5 (62.5)	3 (37.5)			6 (75.0)	2 (25.0)			7 (87.5)	1 (12.5)			7 (87.5)	1 (12.5)			4 (50.0)	4 (50.0)		
Organization of family medicine practice			0.08	0.49 (relatively strong)			0.74	0.08 (negligible)			0.02[a]	0.32 (moderate)			1.0	0.08 (negligible)			0.049[a]	0.35 (moderate)			0.85	0.09 (negligible)
Solo practice	0 (0)	8 (100)			3 (37.5)	5 (62.5)			7 (87.5)	1 (12.5)			7 (87.5)	1 (12.5)			7 (87.5)	1 (12.5)			6 (75.0)	2 (25.0)		
Group practice	1 (1.9)	53 (98.1)			17 (31.5)	37 (68.5)			21 (38.9)	33 (61.1)			46 (85.2)	8 (14.8)			52 (96.3)	2 (3.7)			38 (70.4)	16 (29.6)		
Other	1 (50.0)	1 (50.0)			1 (50.0)	1 (50.0)			1 (50.0)	1 (50.0)			2 (100)	0 (0)			1 (50.0)	1 (50.0)			1 (50.0)	1 (50.0)		

[a] Denotes significant results (p<0.05).

References

1. Rea LM, Parker RA. Designing and conducting survey research: A comprehensive guide. San Francisco: Jossey-Boss; 1992. p. 203.
